# Supplementary material for: A global multi-hazard risk analysis of road and railway infrastructure assets
Source: Nat Commun. 2019 Jun 25;10:2677. doi: 10.1038/s41467-019-10442-3 (PMC6592920; doi:10.1038/s41467-019-10442-3)
Supplement: Supplementary file 1 — Supplementary Information [file 41467_2019_10442_MOESM1_ESM.pdf]

Supplementary information

A global multi-hazard risk analysis of road and railway infrastructure assets

Koks et al.

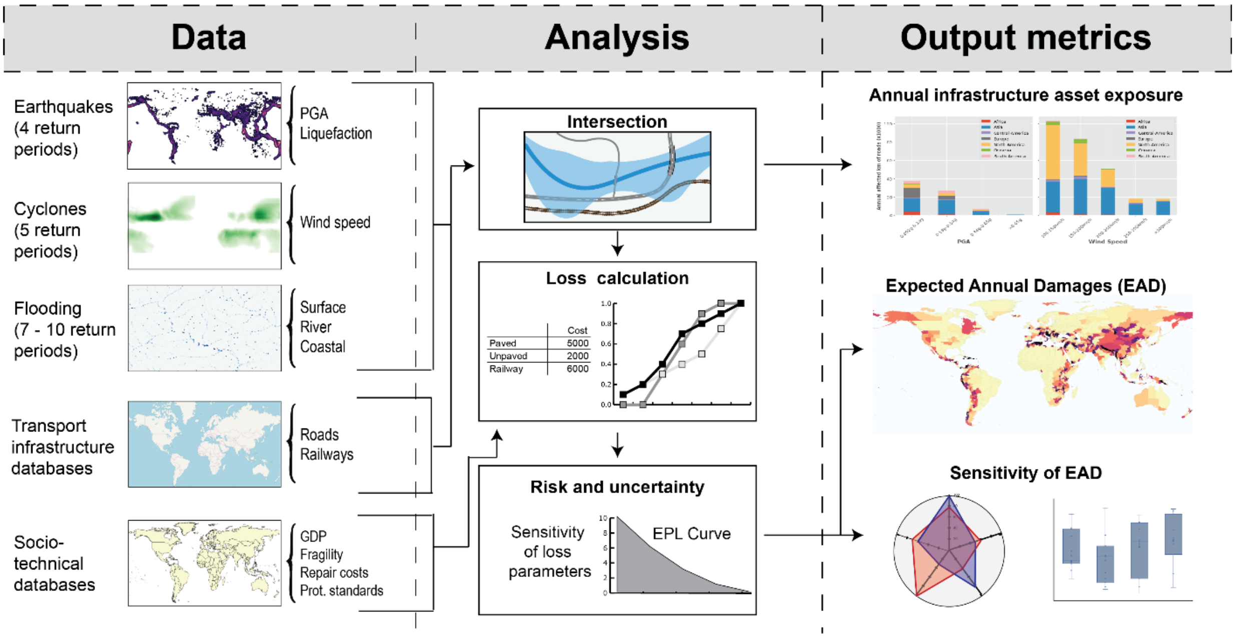

Supplementary Figure 1 | Methodological overview of this study. The leftmost panel presents all data used in this study. The middle panel shows the modelling steps taken to calculate the results. The rightmost panel presents an overview of the different outcomes of the modelling exercise.

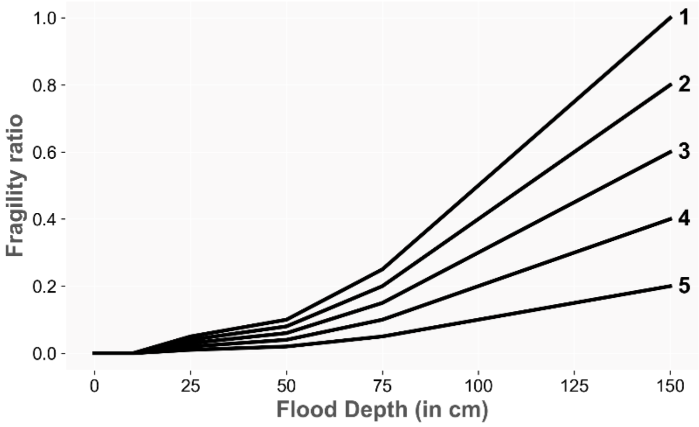

Supplementary Figure 2 | Flood fragility curves for paved roads and railway. Curve #5 adapted from Espinet et al. (2017). Curve 1 – 4 are a multiplication of curve 5 by respectively a factor 2, 3, 4 and 5.

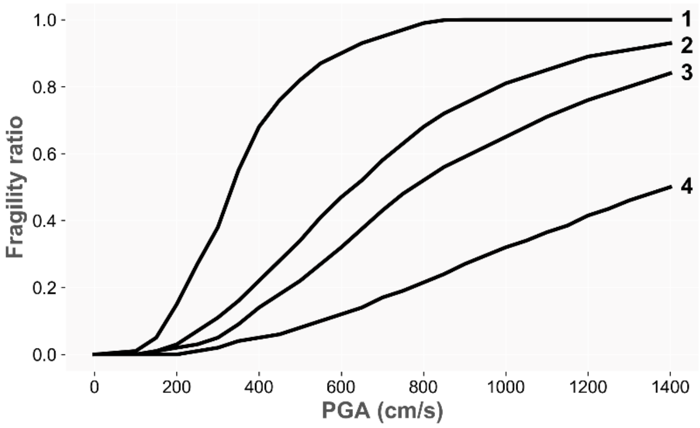

Supplementary Figure 3 | Earthquake fragility curves for road and railway bridges. Curves adapted from Pitalikis (2013)

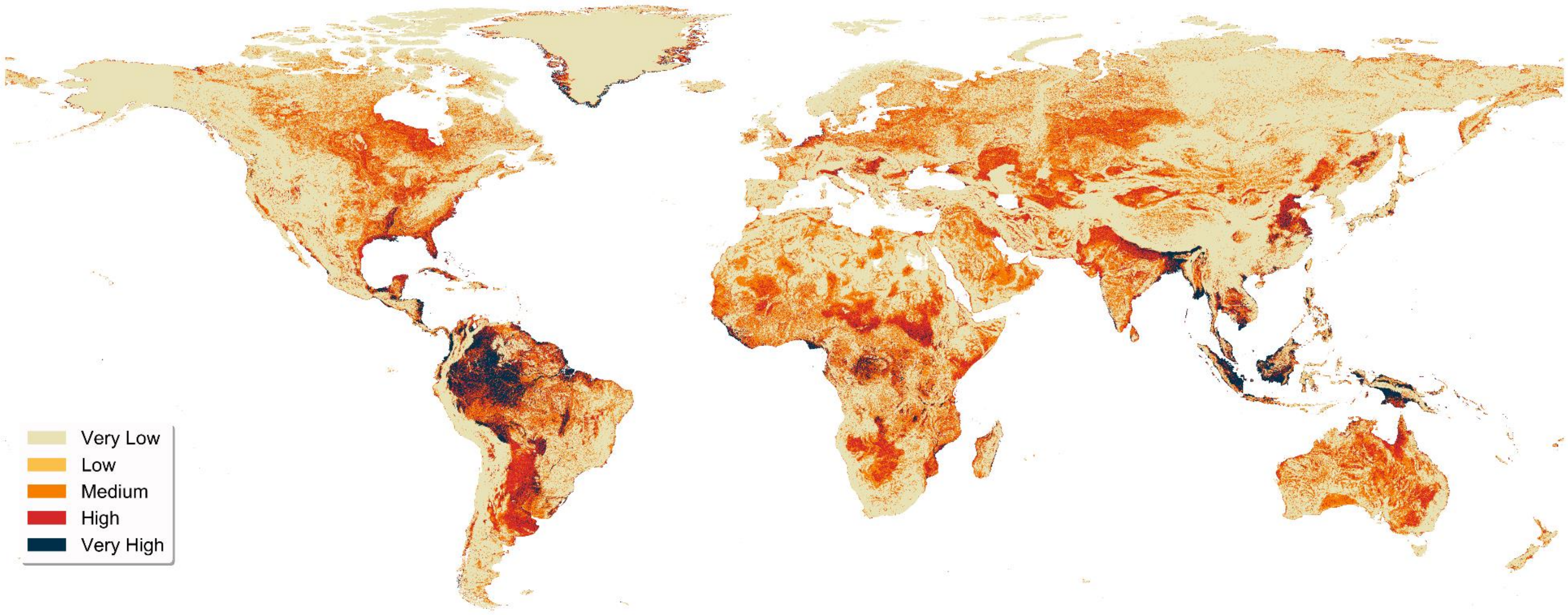

Supplementary Figure 4 | Global Liquefaction Susceptibility Map

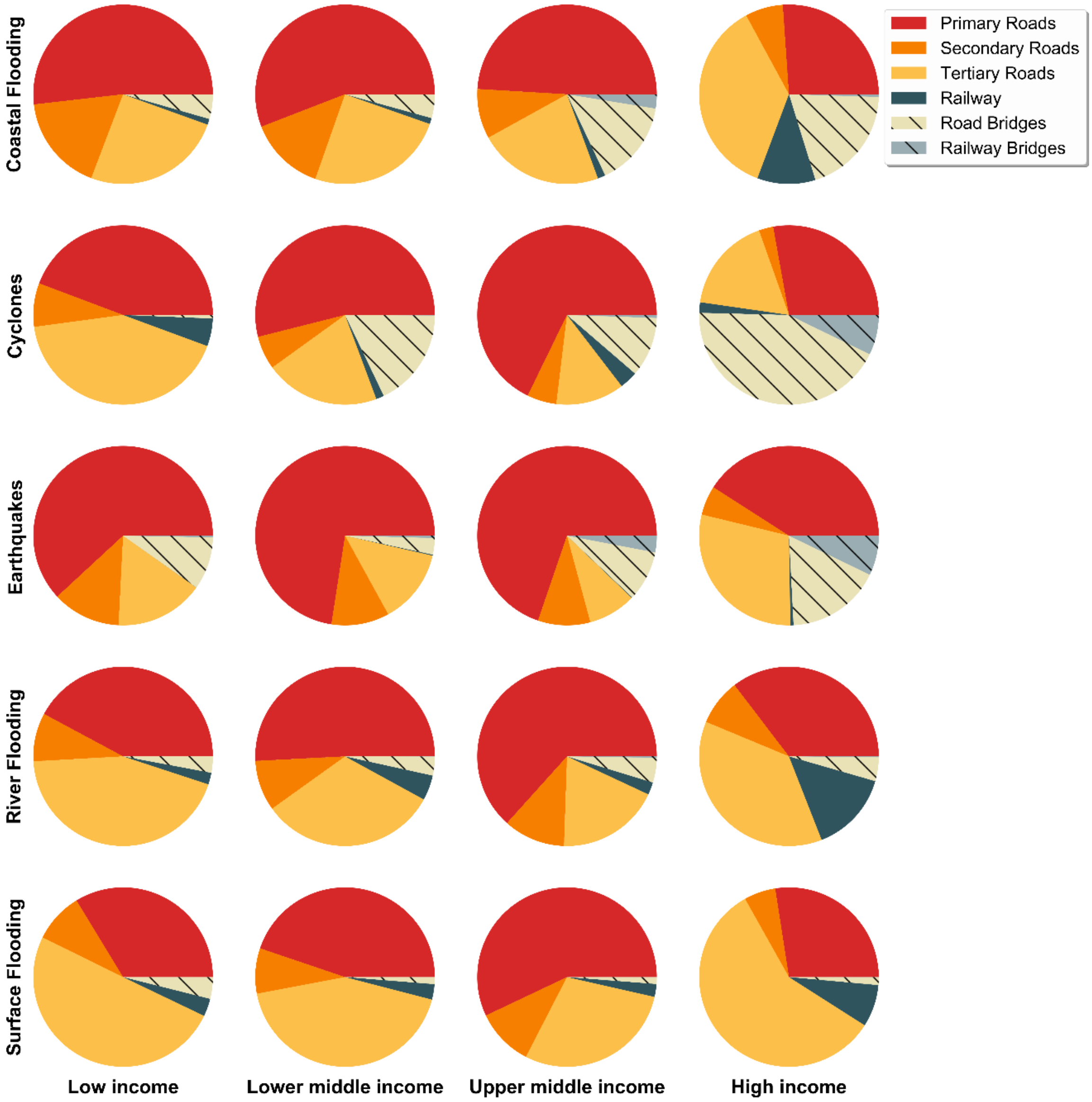

Supplementary Figure 5 | Relative distribution of Expected Annual Damages. The rows represent the different hazards. The columns represent the different income groups.

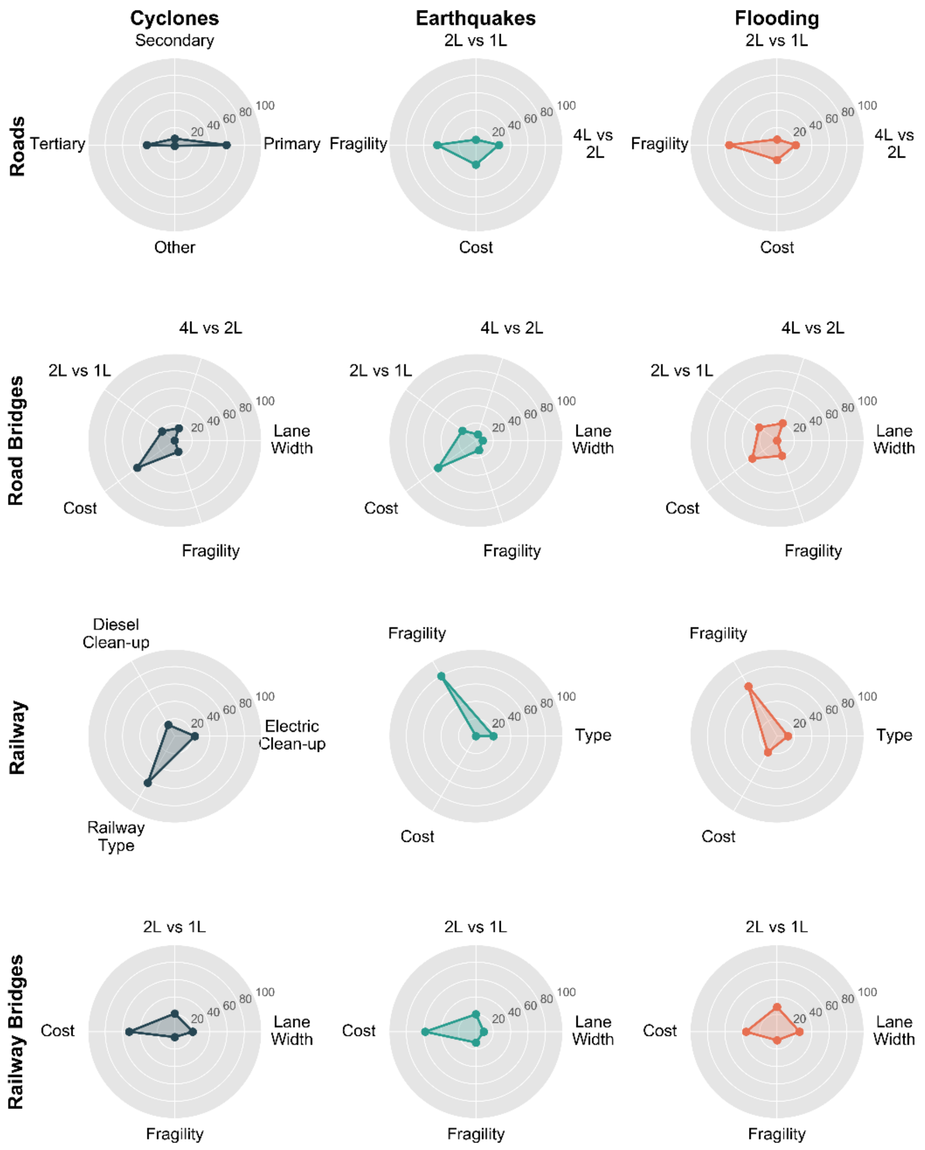

Supplementary Figure 6 | Sensitivity of parameters in the vulnerability analysis. The rows show respectively the parameter sensitivity for roads, road bridges, railway and railway bridges. The columns represent, respectively, tropical cyclones, earthquakes and flooding. All floods are combined, as they are estimated using the same parameters. See **Supplementary Table 1** for an overview of the meaning of all parameters and the variation we apply to the values of each parameter. For cyclone road damage, the road types are representing the clean-up cost for each different road type. *4L vs 2L* indicates the ratio between 2 and 4 lanes, *2L vs 1L* indicate the ratio between 1 and 2 lanes. For railways, *type* represents the parameter which scales between 0 and 1, indicating the percentage of electrified railway (versus diesel).

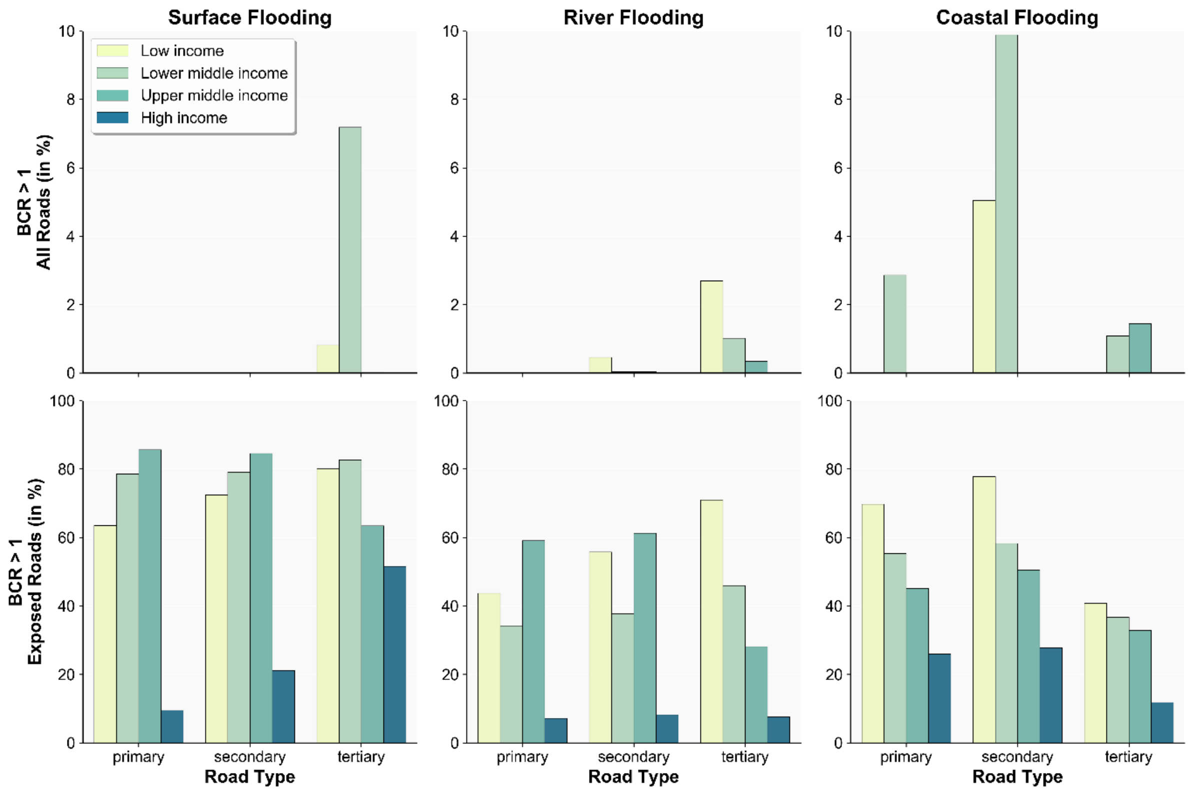

Supplementary Figure 7 | Results of the Cost Benefit Analysis. The upper panels show the percentage of *all* roads that show a positive benefit cost ratio (BCR). The lower panels show the results of the percentage of *exposed* roads that show a positive BCR. Note the different y-axis between the upper and power panels.

Supplementary Table 1 | List of all assumptions taken in this study and their range in the sensitivity analysis

Hazard(s)	Infrastructure	Asset type	Varying parameter	Range	Notes
Earthquake, flooding	Roads	Paved, unpaved	Construction cost	[-25% – +25%]	see Table S8
Earthquake, flooding	Roads	Primary	Lane count	[0 -- 1]	Ratio between 2 and 4 lanes
Earthquake, flooding	Roads	Secondary ,tertiary	Lane count	[0 -- 1]	Ratio between 1 and 2 lanes
Flooding	Roads	Paved roads	Fragility function	[1 – 5]	see Fig S3
Flooding	Roads	Unpaved roads	Fragility function	[1 – 5]	see Fig S3
Earthquakes	Roads	All road assets	Fragility matrix	[1 – 4]	see Table S6
Cyclones	Roads	Primary	Clean-up cost and repair	[5000 – 50000]	USD/km. Assumption based on Escobedo et al. (2009)
Cyclones	Roads	Secondary	Clean-up cost and repair	[1000 – 10000]	USD/km. Assumption based on Escobedo et al. (2009)
Cyclones	Roads	Tertiary	Clean-up cost and repair	[500 – 5000]	USD/km. Assumption based on Escobedo et al. (2009)
Cyclones	Roads	Other	Clean-up cost and repair	[0 – 500]	USD/km. Assuming it will be cleared locally without government intervention
Earthquake, flooding	Railway	Diesel, electrified	Construction cost	[-25% – +25%]	1,000,000 USD/km for electrified, 750,000 USD/km for diesel (Carruthers, 2013)
Earthquake, flooding	Railway	Diesel, electrified	Railway type	[0 -- 1]	Ratio between diesel and electrified railway
Earthquake	Railway	Diesel ,electrified	Fragility matrix	[1 – 4]	see Table S6
Flooding	Railway	Diesel, electrified	Fragility function	[1 – 5]	see Fig S3
Cyclones	Railway	Diesel	Clean-up cost and repair	[5000 – 50000]	Assumption based on Escobedo et al. (2009)
Cyclones	Railway	Electrified	Clean-up cost and repair	[1000 – 10000]	Assumption based on Escobedo et al. (2009)
Earthquake, flooding, cyclones	Bridges	Primary	Lane count	[0 -- 1]	Ratio between 2 and 4 lanes
Earthquake, flooding, cyclones	Bridges	Secondary, tertiary	Lane count	[0 -- 1]	Ratio between 1 and 2 lanes
Earthquake, flooding, cyclones	Bridges	Diesel, electrified	Lane count	[0 -- 1]	Ratio between 1 and 2 lanes
Earthquake, flooding, cyclones	Bridges	All assets	Construction Cost	[-25% – +25%]	See bridge structure costs for values that are varied
Earthquake, flooding, cyclones	Bridges	All assets	Short structure cost	[1237 – 2152]	USD/km[1].
Earthquake, flooding, cyclones	Bridges	All assets	Medium/long structure cost	[914 – 2421]	USD/km[1].
Earthquake, flooding, cyclones	Bridges	All road assets	Lane width	[2.7 – 4.6]	In meters.
Earthquake, flooding, cyclones	Bridges	Diesel, electrified	Lane width	[3 – 5]	In meters.
Flooding	Bridges	Primary	Failure threshold	[700 – 400]	Range of failure threshold, in cm
Flooding	Bridges	Secondary	Failure threshold	[600 – 300]	Range of failure threshold, in cm
Flooding	Bridges	Other	Failure threshold	[500 – 200]	Range of failure threshold, in cm
Flooding	Bridges	Diesel, electrified	Failure threshold	[700 – 400]	Range of failure threshold, in cm
Cyclones	Bridges	Primary	Failure threshold	[400 – 325]	Range of failure threshold, in km/h
Cyclones	Bridges	Secondary	Failure threshold	[375 – 300]	Range of failure threshold, in km/h
Cyclones	Bridges	Other	Failure threshold	[350 – 275]	Range of failure threshold, in km/h
Cyclones	Bridges	Diesel, electrified	Failure threshold	[400 – 325]	Range of failure threshold, in km/h
Earthquakes	Bridges	All assets	Fragility curves	[1 – 4]	see Fig S4

[1] http://www.fdot.gov/structures/StructuresManual/2009january/DesignGuidelines/SDG9.1General.htm

Supplementary Table 2 | List of countries and their income group

Name	ISO3	Income Group
Aruba	ABW	High income
Afghanistan	AFG	Low income
Angola	AGO	Lower middle income
Anguilla	AIA	Upper middle income
Albania	ALB	Upper middle income
Andorra	AND	High income
United Arab Emirates	ARE	High income
Argentina	ARG	High income
Armenia	ARM	Upper middle income
American Samoa	ASM	Upper middle income
Antigua and Barbuda	ATG	High income
Australia	AUS	High income
Austria	AUT	High income
Azerbaijan	AZE	Upper middle income
Burundi	BDI	Low income
Belgium	BEL	High income
Benin	BEN	Low income
Bonaire, Sint Eustatius and Saba	BES	Upper middle income
Burkina Faso	BFA	Low income
Bangladesh	BGD	Lower middle income
Bulgaria	BGR	Upper middle income
Bahrain	BHR	High income
Bahamas	BHS	High income
Bosnia and Herzegovina	BIH	Upper middle income
Belarus	BLR	Upper middle income
Belize	BLZ	Upper middle income
Bermuda	BMU	High income
Bolivia	BOL	Lower middle income
Brazil	BRA	Upper middle income
Barbados	BRB	High income
Brunei	BRN	High income
Bhutan	BTN	Lower middle income
Botswana	BWA	Upper middle income
Central African Republic	CAF	Low income
Canada	CAN	High income
Cocos Islands	CCK	Lower middle income
Switzerland	CHE	High income
Chile	CHL	High income
China	CHN	Upper middle income
Cote d'Ivoire	CIV	Lower middle income
Cameroon	CMR	Lower middle income
Democratic Republic of the Congo	COD	Low income
Republic of Congo	COG	Lower middle income
Cook Islands	COK	Lower middle income
Colombia	COL	Upper middle income
Comoros	COM	Low income
Cape Verde	CPV	Lower middle income
Costa Rica	CRI	Upper middle income
Cuba	CUB	Upper middle income
Curacao	CUW	High income
Christmas Island	CXR	Lower middle income
Cayman Islands	CYM	High income
Cyprus	CYP	High income
Czech Republic	CZE	High income
Germany	DEU	High income
Djibouti	DJI	Lower middle income
Dominica	DMA	Upper middle income
Denmark	DNK	High income
Dominican Republic	DOM	Upper middle income
Algeria	DZA	Upper middle income
Ecuador	ECU	Upper middle income
Egypt	EGY	Lower middle income
Eritrea	ERI	Low income
Western Sahara	ESH	Low income
Spain	ESP	High income
Saint Lucia	LCA	Upper middle income
Liechtenstein	LIE	High income
Sri Lanka	LKA	Lower middle income
Lesotho	LSO	Lower middle income
Lithuania	LTU	High income
Luxembourg	LUX	High income
Latvia	LVA	High income
Saint-Martin	MAF	High income
Morocco	MAR	Lower middle income
Monaco	MCO	High income
Moldova	MDA	Lower middle income
Madagascar	MDG	Low income
Maldives	MDV	Upper middle income
Mexico	MEX	Upper middle income
Marshall Islands	MHL	Upper middle income
Macedonia	MKD	Upper middle income
Mali	MLI	Low income
Malta	MLT	High income
Myanmar	MMR	Lower middle income
Montenegro	MNE	Upper middle income
Mongolia	MNG	Lower middle income
Mozambique	MOZ	Low income
Mauritania	MRT	Lower middle income
Montserrat	MSR	Upper middle income
Martinique	MTQ	Upper middle income
Mauritius	MUS	Upper middle income
Malawi	MWI	Low income
Malaysia	MYS	Upper middle income
Mayotte	MYT	Upper middle income
Namibia	NAM	Upper middle income
New Caledonia	NCL	High income
Niger	NER	Low income
Norfolk Island	NFK	High income
Nigeria	NGA	Lower middle income
Nicaragua	NIC	Lower middle income
Niue	NIU	Upper middle income
Netherlands	NLD	High income
Norway	NOR	High income
Nepal	NPL	Low income
Nauru	NRU	Upper middle income
New Zealand	NZL	High income
Oman	OMN	High income
Pakistan	PAK	Lower middle income
Panama	PAN	High income
Pitcairn Islands	PCN	High income
Peru	PER	Upper middle income
Philippines	PHL	Lower middle income
Palau	PLW	High income
Papua New Guinea	PNG	Lower middle income
Poland	POL	High income
Puerto Rico	PRI	High income
North Korea	PRK	Low income
Portugal	PRT	High income
Paraguay	PRY	Upper middle income
Palestine	PSE	Lower middle income
French Polynesia	PYF	High income
Qatar	QAT	High income
Reunion	REU	Upper middle income
Romania	ROU	Upper middle income
Russia	RUS	Upper middle income
Rwanda	RWA	Low income
Saudi Arabia	SAU	High income
Sudan	SDN	Lower middle income
Senegal	SEN	Low income
Singapore	SGP	High income

Name	ISO3	Income Group
Estonia	EST	High income
Ethiopia	ETH	Low income
Finland	FIN	High income
Fiji	FJI	Upper middle income
Falkland Islands	FLK	High income
France	FRA	High income
Faroe Islands	FRO	High income
Micronesia	FSM	Lower middle income
Gabon	GAB	Upper middle income
United Kingdom	GBR	High income
Georgia	GEO	Lower middle income
Guernsey	GGY	High income
Ghana	GHA	Lower middle income
Gibraltar	GIB	High income
Guinea	GIN	Low income
Guadeloupe	GLP	Upper middle income
Gambia	GMB	Low income
Guinea-Bissau	GNB	Low income
Equatorial Guinea	GNQ	Upper middle income
Greece	GRC	High income
Grenada	GRD	Upper middle income
Greenland	GRL	High income
Guatemala	GTM	Upper middle income
French Guiana	GUF	Lower middle income
Guyana	GUY	Upper middle income
Honduras	HND	Lower middle income
Croatia	HRV	High income
Haiti	HTI	Low income
Hungary	HUN	High income
Indonesia	IDN	Lower middle income
India	IND	Lower middle income
Ireland	IRL	High income
Iran	IRN	Upper middle income
Iraq	IRQ	Upper middle income
Iceland	ISL	High income
Israel	ISR	High income
Italy	ITA	High income
Jamaica	JAM	Upper middle income
Jersey	JEY	High income
Jordan	JOR	Upper middle income
Japan	JPN	High income
Kazakhstan	KAZ	Upper middle income
Kenya	KEN	Lower middle income
Kyrgyzstan	KGZ	Lower middle income
Cambodia	KHM	Lower middle income
Kiribati	KIR	Lower middle income
Saint Kitts and Nevis	KNA	High income
South Korea	KOR	High income
Kuwait	KWT	High income
Laos	LAO	Lower middle income
Lebanon	LBN	Upper middle income
Liberia	LBR	Low income
Libya	LBY	Upper middle income
South Georgia and the South Sandwich Islands	SGS	High income
Svalbard and Jan Mayen	SJM	High income
Solomon Islands	SLB	Lower middle income
Sierra Leone	SLE	Low income
El Salvador	SLV	Lower middle income
San Marino	SMR	High income
Somalia	SOM	Low income
Saint Pierre and Miquelon	SPM	High income
Serbia	SRB	Upper middle income
South Sudan	SSD	Low income
Sao Tome and Principe	STP	Lower middle income
Suriname	SUR	Upper middle income
Slovakia	SVK	High income
Slovenia	SVN	High income
Sweden	SWE	High income
Swaziland	SWZ	Lower middle income
Sint Maarten	SXM	High income
Seychelles	SYC	High income
Syria	SYR	Low income
Turks and Caicos Islands	TCA	High income
Chad	TCD	Low income
Togo	TGO	Low income
Thailand	THA	Upper middle income
Tajikistan	TJK	Low income
Tokelau	TKL	Lower middle income
Turkmenistan	TKM	Upper middle income
Timor-Leste	TLS	Lower middle income
Tonga	TON	Upper middle income
Trinidad and Tobago	TTO	High income
Tunisia	TUN	Lower middle income
Turkey	TUR	Upper middle income
Tuvalu	TUV	Upper middle income
Taiwan	TWN	High income
Tanzania	TZA	Low income
Uganda	UGA	Low income
Ukraine	UKR	Lower middle income
United States Minor Outlying Islands	UMI	High income
Uruguay	URY	High income
United States	USA	High income
Uzbekistan	UZB	Lower middle income
Saint Vincent and the Grenadines	VCT	Upper middle income
Venezuela	VEN	Upper middle income
British Virgin Islands	VGB	High income
Virgin Islands, U.S.	VIR	High income
Vietnam	VNM	Lower middle income
Vanuatu	VUT	Lower middle income
Wallis and Futuna	WLF	Lower middle income
Samoa	WSM	Upper middle income
Kosovo	XKO	Upper middle income
Yemen	YEM	Low income
South Africa	ZAF	Upper middle income
Zambia	ZMB	Lower middle income
Zimbabwe	ZWE	Low income

Supplementary Table 3 | Hazard reclassification scheme

	Earthquake (ShakeMap Intensity and PGA)	Cyclone (Saffir-Simpson)	Flood (cm)
Very low/ no impact	< V (<0.092g)	< 154 km/h	< 25
Low intensity	VI (0.092 – 0.18g)	2 (154-177 km/h)	25 – 50
Medium intensity	VII (0.18 – 0.34g)	3 (178-208 km/h)	50 – 100
High intensity	VIII (0.34-0.65g)	4 (209-251 km/h)	100 – 200
Extreme intensity	> IX (>0.65g)	5 (>252 km/h)	> 200

Supplementary Table 4 | Mapping from OSM road classification to the classification in this study

OSM type	Road Type	OSM type	Road Type
disused	other	cycleway	other
dummy	other	footway	other
planned	other	living_street	tertiary
platform	other	path	other
unsurfaced	other	pedestrian	other
traffic_island	other	primary	primary
razed	other	primary_link	primary
abandoned	other	residential	tertiary
services	other	road	secondary
proposed	other	secondary	secondary
corridor	other	secondary_link	secondary
bus_guideway	other	service	tertiary
bus_stop	other	steps	other
rest_area	other	tertiary	tertiary
yes	other	tertiary_link	tertiary
trail	other	track	other
escape	other	unclassified	tertiary
raceway	other	trunk	primary
emergency_access_point	other	motorway	primary
emergency_bay	other	trunk_link	primary
construction	other	motorway_link	primary
bridleway	other	other	other

Supplementary Table 5 | Overview of total kilometer of infrastructure assets per income group (in million km)

	Railway	Railway Bridges	Road Bridges	Primary roads	Secondary roads	Tertiary roads	Other roads	Total
Low income	0.05	0.0003	0.003	0.27	0.27	2.24	1.26	4.09
Lower middle income	0.36	0.01	0.02	1.02	0.65	5.31	1.32	8.68
Upper middle income	0.92	0.04	0.09	2.35	1.54	9.49	3.65	18.08
High income	1.28	0.03	0.12	2.37	1.92	19.37	10.21	35.30
**Total**	2.61	0.08	0.23	6.01	4.39	36.40	16.44	**66.15**

Supplementary Table 6 | PGA and soil liquefaction damage matrices. The values in brackets represent the different damage ratios that correspond to the combination of the liquefaction band and the PGA level.

	Liquefaction band				
	very high	high	medium	low	very low
**< V (<0.092g)**	0	0	0	0	0
**VI (0.092 – 0.18g)**	[10,20,30,40]	0	0	0	0
**VII (0.18 – 0.34g)**	[25,40,55,70]	[10,20,30,40]	0	0	0
**VIII (0.34-0.65g)**	[40,60,80,100]	[25,40,55,70]	[10,20,30,40]	0	0
**> IX (>0.65g)**	100	[40,60,80,100]	[25,40,55,70]	[10,20,30,40]	0

Supplementary Table 7 | Road and railway flood design standards assumed in this study

Income Group	Primary, Secondary & Railway	Tertiary
High Income	1/50	1/20
Upper Middle Income	1/20	1/10
Lower Middle Income	1/10	1/10
Lower Income	1/10	1/10

Supplementary Table 8 | Road construction cost per WB region in USD

Region	Paved 4 lanes	Paved 2 lanes	Gravel
South Asia	3,570,100	843,120	19,450
Sub-Saharan Africa	3,800,000	932,740	22,780
Middle East & North Africa	2,333,350	665,320	19,350
East Asia & Pacific	4,597,100	1,200,000	38,540
Latin America & Caribbean	4,153,850	1,395,000	37,495
Europe & Central Asia	1,718,350	1,587,900	26,550
Remaining High Income Countries	3,500,000	1,000,000	30,000

Supplementary References

Carruthers, R. (2013). What prospects for transport infrastructure and impacts on growth in southern and eastern Mediterranean countries? Medpro Report No. 3, Medpro.

Escobedo, F. J., Luley, C. J., Bond, J., Staudhammer, C., & Bartel, C. (2009). Hurricane debris and damage assessment for Florida urban forests. *Journal of Arboriculture*, *35*(2), 100.

Espinet, X., Rozenberg, J., Rao, K. S., & Ogita, S. (2018). Piloting the use of network analysis and decision-making under uncertainty in transport operations: preparation and appraisal of a rural roads project in Mozambique under changing flood risk and other deep uncertainties. Washington DC, The World Bank.

Pitilakis, K. D., S. Argyroudis, K. Kakderi, and A. Argyroudi (2013). Systemic seismic vulnerability and risk analysis for buildings, lifeline networks and infrastructures safety gain. SYNER-G synthetic document. JRC Scientific and Policy Reports. doi:10.2788/23242
